# Obesity related metabolic endotoxemia is associated with oxidative stress and impaired sperm DNA integrity

**DOI:** 10.1186/s12610-019-0087-5

**Published:** 2019-05-13

**Authors:** Karma L. Pearce, Amy Hill, Kelton P. Tremellen

**Affiliations:** 1 School of Pharmacy and Medical Sciences, Division of Health Sciences, University of South Australia, Adelaide, South Australia 5001 Australia; 2 Repromed, 180 Fullarton Road, Dulwich, South Australia Australia; 3 Department of Obstetrics Gynaecology and Reproductive Medicine, Flinders University, Bedford Park, South Australia Australia

**Keywords:** Metabolic endotoxemia, LPS, Sperm DNA integrity, Intestinal permeability, Oxidative stress

## Abstract

**Background:**

Obesity is known to be associated with inflammation, oxidative stress and a resulting reduction in sperm DNA integrity. Importantly, obesity is also reported to be associated with an increase in intestinal permeability with the passage of intestinal bacteria into the circulation (metabolic endotoxemia) that triggers a systemic state of inflammation and resultant oxidative stress. Therefore, we hypothesised that this obesity related increase in intestinal permeability and resultant metabolic endotoxemia (ME) may activate inflammation within the male reproductive tract, leading to increased reactive oxygen species production, sperm oxidative stress and a decline in DNA integrity.

**Results:**

Our pilot study of 37 infertile men confirmed a significant positive correlation between body mass index (BMI), increased intestinal permeability (serum zonulin), metabolic endotoxaemia (LBP), sperm DNA oxidative damage (seminal 8 OhDG) and increasing levels of sperm DNA fragmentation (Halosperm). Metabolic endotoxemia was positively correlated with increasing levels of sperm DNA oxidative damage with this relationship remaining significant, even after adjustment for relevant confounders such as age, BMI and days of abstinence. These observations suggest that metabolic endotoxemia and its associated oxidative stress may be a key driver of sperm DNA damage in obese men.

**Conclusion:**

This study confirms a link between obesity, increasing intestinal permeability and endotoxin exposure, and oxidative mediated sperm DNA damage. This warrants further investigation to fully understand the effect of metabolic endotoxemia on male reproductive function which could result in the new therapies to improve male fertility potential.

## Background

Infertility is a significant public health problem affecting 15% of couples worldwide [1]. Male factors are involved in 50% of cases, with paternal obesity being suggested as an underlying cause for both a reduction in sperm count [2–5] and an increase in sperm DNA damage [6], as well as epigenetic changes with possible long term health consequences for the child if conception were to occur [6]. Furthermore, despite the use of assisted reproductive treatment, paternal obesity has been linked in some studies with reduced live birth rates following In Vitro Fertilisation (IVF) treatment, even after controlling for maternal weight [7, 8]. This may be of particular importance as the proportion of overweight or obese males in reproductive age men in the developed world now exceeds 50% and appears to be increasing over time [9].

A recent review by Mushtaq et al. [10] has reported that obese men are more likely to have reduced semen volume, total sperm number and total sperm count resulting in reduced fertility and fecundity. Furthermore, a recent meta-analysis of 52 animal studies examining the impact of a high-fat obesogenic diet also reported that increased adiposity was associated with reduced semen quality, reduced fertilization and a decline in natural fertility [11]. Kahn et al. [12] report that adipose tissue-derived factors, such as adipokines and leptin regulate inflammation and testosterone production, respectively. Increased systemic inflammation results in increased reactive oxygen species (ROS) and in turn sperm DNA fragmentation. Furthermore, increased testicular temperature and inactivity also impairs spermatogenesis. However, the total effect of obesity on hormone levels, semen parameters and sperm DNA integrity, is variable suggesting multiple mechanisms may be in play [12].

It is well recognised that human observational studies can only draw correlations between BMI and male reproductive function which may not be directly causational in nature. Impaired reproductive function in the obese male is likely to be directly related to both his excessive adiposity, but also other potential confounding lifestyle factors such as a diet high in processed foods or deficient in key micro-nutrients or a lack of exercise. One possible link between obesity and reduced fertility rates that has yet to be explored is metabolic endotoxemia (ME). Here the ingestion of large amounts of food, especially fatty food, has been shown to produce an increase in intestinal permeability [13, 14] and the passage of gut bacteria from within the intestinal lumen into the systemic circulation. These predominantly gram negative gut bacteria contain the potent immune stimulant endotoxin (LPS) on their cell wall which activates a protective systemic inflammatory response to help clear the bacterial intrusion. Recent research from our group has shown that ME is positively correlated with inflammation (serum C-reactive protein, IL-6) in obese men, and this inflammatory response is associated with impaired testicular function signified by reduced testosterone production [15, 16]. However, to date the potential link between obesity, intestinal permeability, metabolic endotoxemia, and impaired sperm quality has yet to be investigated.

Our hypothesis that ME may impair spermatogenesis is scientifically plausibility and outlined in detail our GELDING theory (Gut Endotoxin Leading Decline IN Gonadal function) hypothesis paper [17]. Firstly, ME increases systemic inflammation in humans [18] with inflammation being characterised by elevated ROS (reactive oxygen species) production by activated leukocytes that are known to impair sperm function [19], while also initiating a positive feedback inflammatory loop that further potentiates inflammation and oxidative stress [18, 20–25]. While there is a paucity of evidence linking ME with reduced semen quality, there is good evidence linking leaky gut with ME and systemic inflammation [26], plus there is strong evidence linking inflammation with reduced semen quality [27]. Secondly, LPS (the primary endotoxin molecule) is known to directly impair sperm function. Healthy sperm cultured in the presence of LPS exhibit a decrease in motility and an increased production of potentially damaging ROS [22]. Furthermore, in vivo administration of endotoxin to healthy animals has been shown to initiate seminal oxidative stress [28] and impair sperm production and fertility [28–30]. The presence of endotoxin receptors (TLR 4) on sperm membranes, and their ability to initiate an increase in their IL-6 production in response to LPS [31], suggests that sperm are capable of recognising bacterial endotoxin [32]. Furthermore, exposure of a leukocyte -free sperm suspension to LPS in vitro has been reported to directly initiate apoptosis [32], a known trigger for sperm DNA damage. As such, these studies suggest that endotoxin exposure may alter sperm function both directly and indirectly via activation of a leukocyte ROS response.

The aim of this study was to investigate the effect of metabolic endotoxemia (LPS) on sperm production and quality. We hypothesise that increasing endotoxin levels will activate inflammation within the male reproductive tract, leading to increased leukocyte activity and sperm oxidative stress with a resultant decline in sperm DNA quality.

## Methods

### Study cohort

Between February 2017 and June 2017, 45 men aged 18 to 50 years were recruited from a private fertility clinic in South Australia (Repromed) through advertisements. Inclusion criteria was being in an infertile relationship and exclusion criteria were documented inflammatory or infectious disease, primary hypogonadism (Klinefelters Syndrome, cryptorchidism or testicular injury), the consumption of immunosuppressive medication (e.g., nonsteroidal anti-inflammatory drugs (NSAID), corticosteroids or fish oil), supplements that may alter intestinal function (e.g., probiotics, antibiotics in the last 1 month) or any male hormonal therapy (i.e. aromatase inhibitors, clomiphene citrate, human chorionic gonadotropin (hCG) or testosterone). All men were screened for sub-clinical genital tract infection using seminal plasma elastase [33]. This study was approved by the University of South Australia Ethics Committee (approval number 0000036399), with informed written consent being obtained from all participants.

### Study protocol

Unfasted blood and semen samples were provided by all participants between 7:30 am and 11:00 am on the day of testing. Participants were also required to produce a semen sample via masturbation after maintaining an abstinence period of 2–7 days. The semen was analysed within 2 h of collection. Anthropometric measures were also collected; height was measured to the nearest 1 cm (Seca, 216, Germany). Weight and body fat percentage were measured using a bio-electrical impedance scale (Tanita, UM051, Tanita Corporation of America Inc.). Waist circumference was measured from the midpoint of the distance between the top of the iliac crest and the twelfth rib and measured to the nearest 1 cm. Body mass index (BMI) was calculated using the equation body weight (kg)/height (m^2^) and classified using the WHO ranges; underweight (< 18.50 kg/m^2^), normal weight (18.50 kg/m^2^ – 24.99 kg/m^2^), overweight (25.00 kg/m^2^ – 29.99 kg/m^2^) and obese (> 30 kg/m^2^) [34].

### Endotoxin analysis

Metabolic endotoxemia was quantified indirectly by Lipopolysaccaride Binding Protein (LBP) analysis of plasma in duplicate using a dilution of 1:1000 according to the manufacturer’s guidelines (Hycult, Uden, Netherlands), and as previously reported by our group [15, 16]. The minimum detectable concentration of LBP being 4.4 ng/mL. The assay protocol reports no cross-reactivity with non-human LBP.

### Intestinal permeability: Zonulin

Intestinal permeability was investigated by measuring levels of zonulin in plasma in duplicate using a human zonulin ELISA kit according to the manufacturer’s instructions (Crux, Melbourne; Cusabio, USA). This assay reported an inter-assay coefficient of variation of 3% and reference range of 0.156 ng/mL- 40 ng/mL.

### Seminal plasma inflammatory activity markers

Elastase, a marker of neutrophil activity within the male reproductive tract was measured in frozen (−80C) and then thawed seminal plasma in duplicate using the Human Elastase 2 Neutrophil ELISA kit according to the manufacturer’s instructions (Crux, Melbourne). This test had a coefficient of variation of 2% with a reference range of 1.56 ng/mL– 400 ng/mL, with levels exceeding 290 ng/ml suggestive of genito-urinary tract infection/ inflammation [13].

### Seminal oxidative stress: 8-hydroxy-2′-deoxyguanosine (8-OHdG)

The inflammatory marker 8-hydroxy-2′-deoxyguanosine (8OHdG) was also measured in in frozen (−80C) and then thawed seminal plasma in duplicate using a human 8OHdG ELISA kit according to the manufacturer’s instructions (Crux, Melbourne). This assay had a reference range of 2.0 - 800 ng/mL.

### Semen analysis

Total sperm concentration was calculated according to WHO V guidelines [35]. DNA fragmentation was also assessed in duplicate by the same analyst according to the manufacturer’s guidelines (Halosperm G2, Halotech, Spain). Briefly, total sperm motility was calculated after liquefaction. Two hundred spermatozoa were counted and defined as motile or immotile depending on movement classified by WHO [35]. The numbers were recorded and percentage total motility was determined. A morphology slide was prepared, fixed, stained and scored to determine the sperm morphology according to strict WHO specifications, with the percentage of good morphological spermatozoa in 400 sperm reported [35]. The percentage of fragmented DNA was determined after assessment of 400 individual spermatozoa. Within our laboratory high quality sperm samples (e.g.sperm donors) generally have Halosperm DNA damage levels of less than 5%, with patient results above 20% generally being considered abnormal.

### Hormone analysis

Serum was analysed for testosterone, estradiol, follicle stimulating hormone (FSH) and luteinising hormone (LH) using an automated chemiluminescence immunoassay (Cobas 6000 e 601, Roche Diagnostics, USA), with the detectable ranges for each hormone being 18.4–110.10 pmol/L, 0.087–52.0 nmol/L, 0.1-200mIU/mL and 0.2-100mIU/mL respectively.

### Statistical analysis

Statistical analyses were conducted using Statistical Product and Service Solution Software, version 23 (SPSS Inc., Chicago, IL, USA). Data was expressed as mean (+ standard deviation) when normally distributed, or as a median (inter-quartile range) when not normally distributed according to the Shapiro-Wilk test. Correlations were assessed using the Pearson’s method, with log transformation of non-normally distributed data prior to statistical analysis. Where appropriate, adjustments were made for age, smoking status and measures of adiposity.

## Results

### Participant characteristics

While 43 men were assessed for eligibility; 5 were excluded for taking fish oil and 1 for taking antibiotics within the last month. Of the remaining 37 men available for analysis, their average age was 36.9 ± 5.2 years and BMI was 30.2 ± 4.7 kg/m^2^. Only 10.8% of the cohort were of normal BMI, with the remainder being either overweight (BMI 25–29.9, 45.9%) or obese (BMI > 30, 43,2%) [34]. The male fertility potential of the majority of participants was unknown at the time of recruitment. After full assessment the causes of infertility were pure male factor 14 subjects (37.8%), pure female factor 10 subjects (27%), combined male and female factor 6 subjects (16.2%) and unknown cause 7 subjects (19%). The cohort demographic data is outlined in Table 1.

**Table 1:** Participant demographic, endocrine and inflammatory characteristics

Variable	Mean + SD (median, IQR)	Range
Age (years)	36.9 ± 5.2	28.0–46.0
BMI (kg/m^2^)	30.2 ± 4.7	23.3–41.6
Waist circumference (cm)	97.0 (67.0–131.5)	87.0–127.0
% body fat	28.4 ± 8.4	16.1–45.6
Sperm count (million/mL)	54.5 ± 5.5	1.8–204.0
Sperm motility (%)	57.0 (49.0–65.0)	46.0–81.0
Normal sperm morphology (%)	1.1 ± 1.6	3.5–11.0
Sperm DNA fragmentation (%)	14.0 ± 2.4	8.5–33
Seminal elastase (ng/mL)	29.8 ± 12.2	3.0–67.1
Seminal 8OHdG (ng/mL)	7.5 ± 3.1	0.9–90.8
Total testosterone (nmol/L)	15.3 ± 6.2	4.5–27.1
LH (IU/L)	5.1 ± 1.1	2.5–10.2
FSH (IU/L)	3.5 (1.7–5.2)	1.6–13.9
Estradiol (pmo/L)	77.2 ± 8.2	18.4–98.5

Data expressed as mean + SD or median (Inter-Quartile Range), depending on their normal distribution status

*LBP* Lipopolysaccharide Binding Protein. (*n* = 37)

### BMI, intestinal permeability and endotoxin exposure

As anticipated, there was a significant positive correlation between the intestinal permeability marker serum zonulin and BMI (*R* = 0.331, *p* = 0.045). There was also a significant positive relationship between zonulin and the endotoxemia marker LBP (*R* = 0.374, *p* = 0.025*)*. However, no significant relationships were apparent between serum zonulin and markers of male reproductive tract inflammation (elastase), sperm oxidative damage (8 OHdG) or any sperm quality marker.

**Fig. 1:**
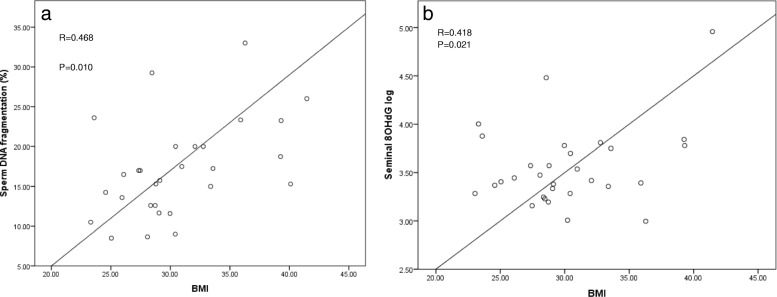
The relationship between BMI and both and sperm DNA damage and seminal 8-hydroxy-2′-deoxyguanosine (8OHdG). **a** Relationship between body mass index (BMI) and sperm DNA damage. **b** Relationship between body mass index (BMI) and seminal 8-hydroxy-2′-deoxyguanosine (8OHdG)

Endotoxemia (LBP) was positively correlated with BMI and % body fat (*R* = 0.375, *p* = 0.024; *R* = 0.407, *p* = 0.017 respectively), (Fig. 1). There were no significant relationships between LBP and total sperm count (*R* = 0.266, *p* = 0.148), sperm morphology (*R* = 0.305, *p* = − 0.102) or sperm motility (*R* = 0.107, *p* = 0.208), with these relationships remaining unaltered after controlling for age, percentage body fat and the number of days abstinence. A key finding was the positive relationship between sperm DNA fragmentation and metabolic endotoxemia (LBP; *R* = 0.460, *p* = 0.021), with this relationship being maintained even after adjustment for age and percentage body fat (*r* = 0.462, *p* = 0.023) (Fig. 1). This positive correlation was also maintained after further adjusting for days abstinence, a key determinant of sperm DNA integrity (*R* = 0.434, *p* = 0.027) [36].

### Sperm DNA quality markers

Sperm DNA fragmentation was also positively associated with all measures of adiposity (BMI; *R* = 0.468, *p* = 0.010, % body fat; *R* = 0.512, *p* = 0.006, waist circumference; *R* = 0.460, *p* = 0.012) (Fig. 1).

**Fig. 2:**
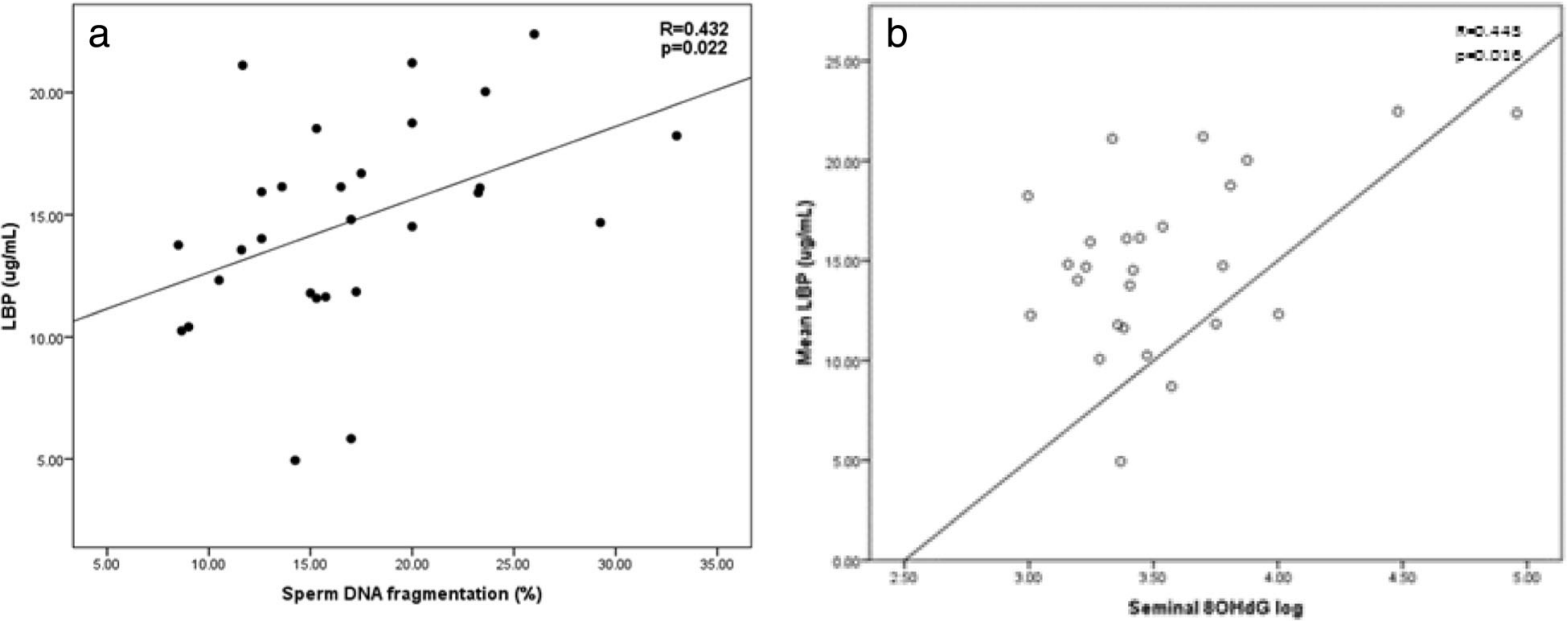
The effect of LBP on both seminal DNA fragmentation and seminal 8-hydroxy-2′-deoxyguanosine (8OHdG). **a** Relationship between an indirect measure of endotoxin (LBP) and seminal DNA fragmentation. **b** Relationship between an indirect measure of endotoxin (LBP) and seminal 8-hydroxy-2′-deoxyguanosine (8OHdG)

As expected, seminal 8OHdG, a marker of seminal oxidative stress was significantly positively correlated with BMI (*R* = 0.418, *p* = 0.021). A negative relationship between sperm motility and seminal 8OHdG (following adjustment for sperm number) was also observed (*R* = -0.624, *p* = 0.002). Conversely, a significant positive relationship was observed between endotoxin exposure (LBP) and seminal 8OHdG (R = 0.440, *p* = 0.016), confirming a link between endotoxemia and oxidative stress (Fig. 2). Seminal 8OHdG was also strongly correlated with BMI (*R* = 0.418, *p* = 0.021) (Fig. 2).

### Hormones

As expected, serum testosterone was inversely correlated with BMI, body fat percentage and waist circumference, with strong effect sizes for each (*R* = -0.623, *p* < 0.001; *R* = -0.643, *p* < 0.001; *R* = -0.677, *p* < 0.001 respectively). All correlations were maintained when adjusted for age (*p* < 0.001 for all variables) (Table 2). Furthermore, FSH was inversely correlated with BMI (*R* = − 0.373, *p* = 0.011). Serum LH and estrogen were not correlated with any measures of adiposity (*p* > 0.05 for all).

**Table 2:**
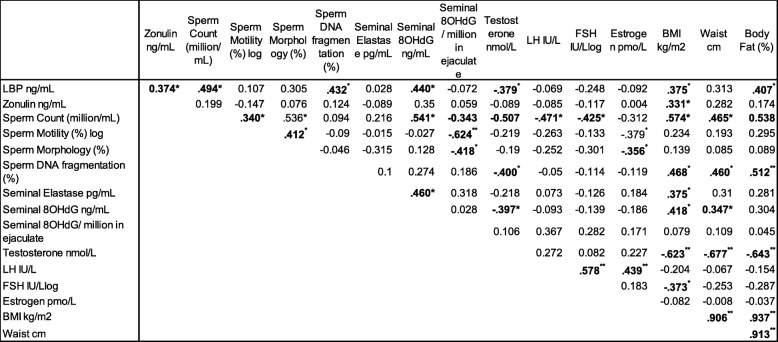
Correlation matrix

Statistical analysis using the Pearson correlation test. All values represent correlation coefficient value (r), with those reaching statistical significance (*p* < 0.05) indicated in bold type

*LH* Luteinising hormone, *FSH* follicle stimulating hormone, *LPS* Lipopolysaccaride

## Discussion

Our research team has recently demonstrated that experimental administration of low-dose endotoxin (0.8 ng *Escherichia coli* O113:H10/kg body weight) results in a rapid decline in testosterone production in healthy reproductive age men [16], thereby directly confirming the capacity of bacterial endotoxin to impair testicular function. However, the results of this pilot study are the first to identify a significant positive correlation between bacterial endotoxin exposure (LBP) and impaired sperm DNA integrity, with this relationship remaining significant even after adjusting for relevant confounders such as age, BMI and duration of abstinence. The results of our study give some insight into a potential mechanism for the increased levels of sperm DNA damage seen in obese men. In summary we believe that obesity and its associated poor diet causes an increase in intestinal permeability that allows bacteria to transmigrate from the intestinal lumen into the circulation (metabolic endotoxemia), resulting in activation of an inflammatory response. This endotoxin elicited inflammation spills over into the male reproductive tract where it increases leukocytes production of ROS, leading to sperm DNA oxidative attack and a decline in sperm DNA integrity.

Zonulin, a protein dynamically produced by the intestinal epithelium and liver that reversibly regulates intestinal permeability by modulating intercellular tight junctions and is generally regarded as a useful non-invasive surrogate marker of intestinal permeability. In the obese state there is an increase in the basal production of zonulin and a resultant decrease in expression of two epithelial tight junction proteins; occluding and zonula occludens-1 [37] that then enables bacteria to translocate between the intestinal lumen and the bloodstream [38], challenging the immune system to [13, 22, 23].

Although the mechanism for the increase in circulating zonulin in obesity is not fully eluded, it is proposed that inflammatory cytokines such as TNFα, and IL-6 produced by adipose tissue may trigger zonulin release, further increasing intestinal permeability, allowing more LPS into circulation. This process highlights the positive feedback loop relationship between obesity, intestinal permeability and metabolic endotoxemia [39, 40]. Radd et al. [41] have recently reviewed the effect of obesity on sperm parameters, although some discrepancies were noted, overall these studies indicated that overweight and obesity in male were associated with an increased percentage of sperm with DNA fragmentation and abnormal mitochondrial membrane potential. In agreement with previous studies in obese men, our study confirmed that serum zonulin was positively associated with both obesity (*R* = 0.331, *p* = 0.045) [39] and metabolic endotoxemia (*R* = 0.0374, *p* = 0.025) [40, 42]. However, no direct significant relationship was observed between intestinal permeability (zonulin) and any marker of sperm quality. However, this lack of significance does not exclude intestinal permeability as cause of reproductive impairment, particular given that serum zonulin was correlated with endotoxemia and endotoxemia with sperm oxidative DNA damage. Furthermore, the trans-mucosal passage of gut bacteria occurs by two pathways [13, 38]. An inter-cellular passage of bacteria through tight junctions between epithelial cells regulated by zonulin and the trans-cellular passage of LPS fragments taken up by bile acid micelles and carried across the intestinal cell membrane independent of zonulin/ ZO action [37]. As LBP measures the net impact of inter and trans-cellular gut bacterial transmigration, we now believe that this may explain why we did not see any direct correlations between zonulin and sperm quality parameters, yet did between LBP (average of days endotoxin exposure) and sperm quality. Furthermore, plasma zonulin undergoes dynamic changes in plasma levels during the day in response to everyday triggers such as psychological stress [43] and food [44], potentially confounding direct associations between this rapidly fluctuating variable and sperm production with much longer time lines.

This study is the first to report positive association between sperm DNA fragmentation with increased endotoxin exposure (*R* = 0.461, *p* = 0.022) even after adjustment for age, smoking status and measures of adiposity, thereby confirming our hypothesis that the presence of endotoxin in systemic circulation can potentially effect sperm quality. Furthermore, there was a significant positive correlation between endotoxemia and sperm DNA oxidative damage, plus markers of adiposity and 8 OHdG levels [5]. We interpret these findings as suggesting that obesity is associated with endotoxin initiated inflammation and the production of ROS by activated leukocytes that then oxidatively damage sperm DNA integrity. Previous work from our group has shown that low-dose endotoxin administration in healthy men produces an increase in serum inflammatory cytokines such as interleukin-6 (IL-6) and tumour necrosis factor-α (TNF-α), both known to activate leukocyte ROS production [16, 45]. Sperm DNA fragmentation is generally accepted to be related to either ROS damage, apoptosis or physiological “nicks” generated during DNA packaging/ proamination. We propose that endotoxin exposure may impair sperm DNA integrity via two mechanisms. Firstly, an indirect mechanism mediated by activation of male reproductive tract leukocytes releasing ROS (oxidative stress). Secondly, a direct mechanism by which LPS may bind to sperm TLR4 receptors and initiate apoptosis, as has been shown in vitro in human and murine sperm [38]. As we did not directly measure apoptosis (annexin V or similar markers) this second apoptotic mechanism of sperm DNA damage is still theoretical only.

Pilatz et al. [46] showed that systemic inflammation related to obesity (increase CRP and IL-6) did not translate into an increase in seminal plasma cytokines, or an increase in seminal plasma elastase, a result also in agreement with our own findings. This suggest that obesity related systemic inflammation may not reach the male reproductive tract, or that LPS initiated localised inflammation in the epididymis and testis may not produce detectable changes in seminal plasma cytokines or elastase, yet still mediate damage to sperm by initiating localised oxidative stress.

Our results suggest that in the context of obesity related oxidative DNA damage, a potential alternative approach to the traditional antioxidant approach may be to administer therapies that fortify intestinal barrier function, reducing subsequent metabolic endotoxemia and inflammation and its associated sperm oxidative damage. One potential approach would be the use of probiotics which have been reported to improve intestinal barrier function, reduce metabolic endotoxemia and inflammation [47]. Several animal studies [48] and one recent human RCT [49] have reported that probiotics can indeed boost sperm quality and testosterone levels. We have postulated that this beneficial effect is mediated by reduction in metabolic endotoxemia [50].

We acknowledge several potential weaknesses in our study. Firstly, western style diets, excessive alcohol intake and smoking are all known to increase systemic inflammation [26]. While this study controlled for the 3 men who smoked [51] and excluded men consuming excessive alcohol, it failed to control for diet. Future studies should control for the influence of inflammatory dietary patterns known to exacerbate metabolic endotoxaemia such as high fat meals [11]. Secondly, this is only a pilot study of 37 men. The recruitment of a larger sample size is likely to produce more statistically rigorous results. Thirdly, while recent meta-analysis show relationships between BMI and DNA fragmentation [44, 52], other studies report conflicting results (reviewed in [53]), which the authors report may be due to the variability in study populations (fertile/ subfertile/ general population), samples size and the heterogeneity of the sperm DNA integrity assays used (TUNEL assay, sperm chromatin structure assay (SCSA), flow cytometry, comet assay method). In this study the Halosperm G2 method was used to assess DNA fragmentation and a number of steps were taken to ensure the reproducibility and accuracy of this analysis. These included earlier calibration against the TUNEL method of analysis, sample analysis within 2 h of receipt, reduction in inter-assay variability by having a single technician (author 2) perform all the analysis and the use of a clinical pathology accredited laboratory. Previous studies within our laboratory have shown very good correlation between TUNEL and Halosperm methods of assessing sperm DNA integrity (*R*^*2*^ = 0.872, *p* < 0.001), plus minimal inter-observer variability, especially for the the Halosperm test. A recent review has also concluded that Halosperm test is a valid assessment of both sperm DNA integrity and male fertility potential [54].

Fourthly, there is presently significant uncertainty regarding the impact of sperm DNA fragmentation on both natural and ART assisted fertility. Some of this may be related to differences in tests performed, the processing of sperm before testing (assessment of neat semen or swim-up processed samples) and the fact that tests of sperm DNA integrity are destructive and as such we can never directly analyse a correlation between the fertilising sperms DNA integrity and pregnancy outcome.

Finally, we acknowledge that our observational cohort was recruited from a reproductive clinic, and while we excluded men with obvious primary testicular failure, it is possible that the semen parameters and hormone levels detected in this study may not fully represent those of the general population. Additionally, differences in the genetic backgrounds of various ethnic populations and epigenetics should also be considered [55]. It is also possible that hormone levels were influenced by sleep disturbances [56] or endocrine disruptors [57] which was not assessed in our study. It is also possible that participant endotoxin levels were influenced psychologically as a result of the stresses of undergoing fertility treatment [58, 59].

## Conclusions

The results of this study suggest for the first time a link between obesity induced by ME, increasing intestinal permeability and endotoxin levels, and oxidative mediated sperm DNA damage. Although we were unable to detect a direct relationship was between intestinal permeability (plasma zonulin) and any marker of sperm quality, we believe this reflects the fact that zonulin mirrors only changes in para-cellular intestinal permeability, not trans-cellular passage of gut endotoxin mediated by fat and bile micelles. These results are important as they suggest that new therapies directed at enhancing intestinal barrier function may be able to reduce obesity related inflammation oxidative stress, thereby boosting sperm quality.

## Abbreviations

8-OHdG: 8-hydroxy-2′ -deoxyguanosine
BMI: Body mass index
CRP: C-reactive protein
FSH: Follicle-stimulating hormone
hCG: Human chorionic gonadotropin
IL-6: Interleukin 6
IVF: In Vitro Fertilisation
LBP: Lipopolysaccharide binding protein
*LH*: *Luteinizing hormone*
LPS: *Lipopolysaccharide*
ME: Metabolic endotoxemia
NSAID: Nonsteroidal anti-inflammatory drugs
RCT: Randomised control trial
ROS: Reactive oxygen species
TNFalpha: Tumor necrosis factor
WHO: World health organisation

## References

[1] Mascarenhas MN, Flaxman SR, Boerma T, Vanderpoel S, Stevens GA (2012). National, regional, and global trends in infertility prevalence since 1990: a systematic analysis of 277 health surveys. PLoS Med.

[2] Sermondade N, Faure C, Fezeu L, Shayeb AG, Bonde JP, Jensen TK (2013). BMI in relation to sperm count: an updated systematic review and collaborative meta-analysis. Hum Reprod Update.

[3] Chavarro JE, Toth TL, Wright DL, Meeker JD, Hauser R (2010). Body mass index in relation to semen quality, sperm DNA integrity and serum reproductive hormone levels among men attending an infertility clinic. Fertil Steril.

[4] Kort HI, Massey JB, Elsner CW, Mitchell-Leef D, Shapiro DB, Witt MA (2006). Impact of body mass index values on sperm quantity and quality. J Androl.

[5] Tunc O, Bakos HW, Tremellen K (2011). Impact of body mass index on seminal oxidative stress. Andrologia..

[6] Dupont C, Faure C, Sermondade N, Boubaya M, Eustache F, Clément P (2013). Obesity leads to higher risk of sperm DNA damage in infertile patients. AJA..

[7] Bakos HW, Henshaw RC, Mitchell M, Lane M (2011). Paternal body mass index is associated with decreased blastocyst development and reduced live birth rates following assisted reproductive technology. Fertil Steril.

[8] Taha EA, Sayed SK, Gaber HD, Abdel Hafez HK, Ghandour N, Zahran A (2016). Does being overweight affect seminal variables in fertile men?. Reprod BioMed Online.

[9] Australian Bureau of Statistics. 4364.0.55.001 - National Health Survey: first results, 2017–18. http://www.abs.gov.au/ausstats/abs@.nsf/mf/4364.0.55.001. Accessed 1 Dec 2018

[10] Mushtaq R, Pundir J, Achilli C, Naji O, Khalaf Y, El-Toukhy T (2018). Effect of male body mass index on assisted reproduction treatment outcome: an updated systematic review and meta-analysis. Reprod BioMed Online.

[11] Crean AJ, Senior AM. High-fat diets reduce male reproductive success in animal models: a systematic review and meta-analysis. Obes Rev. Epub ahead of print. https://www.urotoday.com/recent-abstracts/urologic-oncology/investigative-urology/110327-high-fat-diets-reduce-male-reproductive-success-in-animal-models-a-systematic-review-and-meta-analysis.html. 10.1111/obr.12827 30756459

[12] Kahn BE, Brannigan RE (2017). Obesity and male infertility. Curr Opin Urol.

[13] Genser L, Aguanno D, Soula HA, Dong L, Trystram L, Assmann K (2018). Increased jejunal permeability in human obesity is revealed by a lipid challenge and is linked to inflammation and type 2 diabetes. J Pathol.

[14] Vors C, Pineau G, Drai J, Meugnier E, Pesenti S, Laville M (2015). Postprandial Endotoxemia linked with chylomicrons and lipopolysaccharides handling in obese versus lean men: a lipid dose-effect trial. J Clin Endocrinol Metab.

[15] Tremellen K, Mcphee N, Pearce K (2017). Metabolic endotoxaemia related inflammation is associated with hypogonadism in overweight men. Basic Clin Androl.

[16] Tremellen K, McPhee N, Pearce K, Benson S, Schedlowski M, Engler H (2018). Endotoxin-initiated inflammation reduces testosterone production in men of reproductive age. Am J Physiol Endoc M.

[17] Tremellen K (2016). Gut endotoxin leading to a decline in gonadal function (GELDING) - a novel theory for the development of late onset hypogonadism in obese men. Basic Clin Androl..

[18] Copeland S, Warren HS, Lowry SF, Calvano SE, Remick D, The I (2005). Acute inflammatory response to endotoxin in mice and humans. Clin Diagn Lab Immunol.

[19] Tremellen K (2008). Oxidative stress and male infertility—a clinical perspective. Hum Reprod Update.

[20] Sarkar O, Bahrainwala J, Chandrasekaran S, Kothari S, Mathur P, Agarwal A (2010). Impact of inflammation on male fertility. Frontiers in bioscience (Elite edition).

[21] Buch JP, Kolon TF, Maulik N, Kreutzer DL, Das DK (1994). Cytokines stimulate lipid membrane peroxidation of human sperm. Fertil Steril.

[22] Martínez P, Proverbio F, Camejo MI (2007). Sperm lipid peroxidation and pro-inflammatory cytokines. AJA..

[23] Agarwal A, Aitken RJ, Alvarez JG. Studies on Men's Heahlth and fertility: Humana press; 2012.

[24] Qian L, Shi Q, Gu Y, Song J, Zhou M, Hua M (2012). The relationship between IL-17 and male infertility: semen analysis. Afr J Microbiol Res.

[25] Fraczek M, Sanocka D, Kamieniczna M, Kurpisz M (2008). Proinflammatory cytokines as an intermediate factor enhancing lipid sperm membrane peroxidation in in vitro conditions. J Androl.

[26] Cani PD, Amar J, Iglesias MA, Poggi M, Knauf C, Bastelica D (2007). Metabolic endotoxemia initiates obesity and insulin resistance. Diabetes..

[27] Azenabor A, Ekun AO, Akinloye O (2015). Impact of inflammation on male reproductive tract. J Reprod Infertil.

[28] Aly HAA, El-Beshbishy HA, Banjar ZM (2012). Mitochondrial dysfunction induced impairment of spermatogenesis in LPS-treated rats: modulatory role of lycopene. Eur J Pharmacol.

[29] Collodel G, Moretti E, Brecchia G, Kuželová L, Arruda J, Mourvaki E (2015). Cytokines release and oxidative status in semen samples from rabbits treated with bacterial lipopolysaccharide. Theriogenology..

[30] Wang H, Yang L-L, Hu Y-F, Wang B-W, Huang Y-Y, Zhang C (2014). Maternal LPS exposure during pregnancy impairs testicular development, steroidogenesis and spermatogenesis in male offspring. PloS One.

[31] Urata K, Narahara H, Tanaka Y, Egashira T, Takayama F, Miyakawa I (2001). Effect of endotoxin-induced reactive oxygen species on sperm motility. Fertil Steril.

[32] Okazaki T, Mihara T, Shimada M, Ikeda C, Fujita Y, Negishi H (2011). Toll-like receptors (TLR) 2 and 4 on human sperm recognize bacterial endotoxins and mediate apoptosis. Hum Reprod.

[33] Zorn B, Virant-Klun I, Meden-Vrtovec H (2000). Semen granulocyte elastase: its relevance for the diagnosis and prognosis of silent genital tract inflammation. Hum Reprod.

[34] World health organisation. BMI Classification Geneva 2006. http://apps.who.int/bmi/index.jsp?introPage=intro_3.html. Accessed 1 Aug 2017

[35] World health organisation (2010). WHO laboratory manual for the examination and processing of human semen.

[36] Bakos HW, Thompson JG, Feil D, Lane M (2008). Sperm DNA damage is associated with assisted reproductive technology pregnancy. Int J Androl.

[37] Fasano A (2017). Gut permeability, obesity, and metabolic disorders: who is the chicken and who is the egg?. Am J Clin Nutr.

[38] Held Hales K, Diemer T, Ginde S, Shankar BK, Roberts M, Bosmann HB (2000). Diametric effects of bacterial endotoxin lipopolysaccharide on adrenal and Leydig cell steroidogenic acute regulatory protein. Endocrinology..

[39] Moreno-Navarrete JM, Sabater M, Ortega F, Ricart W, Fernández-Real JM (2012). Circulating Zonulin, a marker of intestinal permeability, is increased in association with obesity-associated insulin resistance. PLoS One.

[40] Mokkala K, Pellonperä O, Röytiö H, Pussinen P, Rönnemaa T, Laitinen K (2017). Increased intestinal permeability, measured by serum zonulin, is associated with metabolic risk markers in overweight pregnant women. Metab.

[41] Raad G, Hazzouri M, Bottini S, Trabucchi M, Azoury J, Grandjean V (2017). Paternal obesity: how bad is it for sperm quality and progeny health?. Basic Clin Androl.

[42] Żak-Gołąb A, Kocełak P, Aptekorz M, Zientara M, Juszczyk Ł, Martirosian G, et al. Gut microbiota, microinflammation, metabolic profile, and zonulin concentration in obese and normal weight subjects. Int J Endocrinol. 2013;2013. 10.1155/2013/674106. 10.1155/2013/674106 PMC3732644 23970898

[43] Linninge C, Jönsson P, Bolinsson H, Önning G, Eriksson J, Johansson G (2018). Effects of acute stress provocation on cortisol levels, zonulin and inflammatory markers in low- and high-stressed men. Biol Psychol.

[44] Mokkala K, Eerola E, Munukka E, Röytiö H, Laitinen K, Ekblad U (2016). Gut microbiota richness and composition and dietary intake of overweight pregnant women are related to serum zonulin concentration, a marker for intestinal permeability. J Nutr.

[45] Rato L, Alves MG, Cavaco JE, Oliveira PF (2014). High-energy diets: a threat for male fertility?. Obes Rev.

[46] Pilatz A, Hudemann C, Wolf J, Halefeld I, Paradowska-Dogan A, Schuppe H-C (2017). Metabolic syndrome and the seminal cytokine network in morbidly obese males. Andrology..

[47] Markowiak P, Śliżewska K (2017). Effects of probiotics, prebiotics, and synbiotics on human health. Nutrients..

[48] Poutahidis T, Springer A, Levkovich T, Qi P, Varian BJ, Lakritz JR (2014). Probiotic microbes sustain youthful serum testosterone levels and testicular size in aging mice. PLoS One.

[49] Maretti C, Cavallini G (2017). The association of a probiotic with a prebiotic (Flortec, Bracco) to improve the quality/quantity of spermatozoa in infertile patients with idiopathic oligoasthenoteratospermia: a pilot study. Andrology..

[50] Tremellen K, Pearce K (2017). Probiotics to improve testicular function– a comment on mechanism of action and therapeutic potential of probiotics beyond reproduction. Andrology..

[51] Wang HJ, Zakhari S, Jung MK (2010). Alcohol, inflammation, and gut-liver-brain interactions in tissue damage and disease development. WJG..

[52] Campbell JM, Lane M, Owens JA, Bakos HW (2015). Paternal obesity negatively affects male fertility and assisted reproduction outcomes: a systematic review and meta-analysis. Reprod BioMed Online.

[53] JBA O, Petersen CG, Mauri AL, Vagnini LD, Renzi A, Petersen B (2018). Association between body mass index and sperm quality and sperm DNA integrity. A large population study. Andrologia.

[54] Ribas-Maynou J, García-Peiró A, Fernández-Encinas A, Abad C, Amengual MJ, Prada E (2013). Comprehensive analysis of sperm DNA fragmentation by five different assays: TUNEL assay, SCSA, SCD test and alkaline and neutral comet assay. Andrology-US..

[55] Donkin I, Barrès R (2018). Sperm epigenetics and influence of environmental factors. Mol Metab.

[56] Cho JW, Duffy JF. Sleep, sleep disorders, and sexual dysfunction. World J Mens Health. 2018;36. 10.5534/wjmh.180045. 10.5534/wjmh.180045 PMC6704301 30209897

[57] Heindel JJ, Blumberg B, Cave M, Machtinger R, Mantovani A, Mendez MA (2017). Metabolism disrupting chemicals and metabolic disorders. Reprod Toxicol.

[58] de Punder K, Pruimboom L (2015). Stress induces Endotoxemia and low-grade inflammation by increasing barrier permeability. Front Immunol.

[59] Wiweko B, Anggraheni U, Elvira SD, Lubis HP. Distribution of stress level among infertility patients. Middle East Fertil Soc J. 2017;22(2):145–48

